# Hyperacusis questionnaire and event-related potential correlation in migraine patients

**DOI:** 10.1038/s41598-024-65014-3

**Published:** 2024-06-19

**Authors:** Liu Qi, Zhang Jilei, Yu Lisheng, Jing Yuanyuan

**Affiliations:** Department of Otorhinolaryngology-Head and Neck Surgery, Peking University People’s Hospital, Beijing, 100044 China

**Keywords:** Migraine, Auditory hypersensitivity, Event-related potentials, Auditory system, Cognitive neuroscience, Emotion

## Abstract

This study aims to investigate auditory hypersensitivity and cortical function in migraine patients using the Hyperacusis Questionnaire and the Event-Related Potential (ERP) technique. The study analyzes alterations in the latency and amplitude of the event-related potentials MMN and P300 components. The findings contribute to a better understanding of the physiological relationship between migraine and auditory hypersensitivity. Seventeen migraine patients were admitted to the outpatient clinic of the Department of Otorhinolaryngology-Head and Neck Surgery at Peking University People’s Hospital from June 2023 to September 2023. Nineteen matched healthy subjects were also selected. All participants underwent the pure tone audiometry and the auditory brainstem response test to determine hearing thresholds, the Hyperacusis Questionnaire, the Tinnitus Handicap Inventory, and an ERP examination. The Oddball classical paradigm was used as the stimulation task, and electroencephalography signals were recorded synchronously. The scores of the Hyperacusis Questionnaire, latency and amplitude of MMN and P300 component were compared between the migraine group and the control group, and their correlation was analyzed. The latency of MMN at the Fz and Cz sites in migraine patients was significantly shorter than that in the control group (*P* < 0.05), and the amplitudes were significantly higher than those in the control group (*P* < 0.05). The variances in latency and amplitude of P300 at Cz and Pz sites in migraine patients were not statistically significant when compared with the control group. (*P* > 0.05). The Hyperacusis Questionnaire was negatively correlated with MMN latency, with a correlation coefficient of − 0.374 (*P* = 0.025), and positively correlated with MMN amplitude, with a correlation coefficient of 0.378 (*P* = 0.023). There was no significant similarity between the Hyperacusis Questionnaire and P300 latency and amplitude (*P* > 0.05). Overall, auditory hypersensitivity was enhanced in individuals with migraines compared to healthy individuals, leading to faster information processing, while there may be less impairment in cognitive function.

## Introduction

Migraine is a chronic episodic neurological disorder characterized by episodes of moderate to severe headaches accompanied by nausea and vomiting, reversible neurological symptoms, and systemic symptoms^1^. The prevalence of migraine in the population is high, with an estimated 1.04 billion people worldwide suffering from migraine. The age-standardized prevalence is 14.4%, with 18.9% in women and 9.8% in men^2^. Migraine often experiences auditory hypersensitivity, mainly known as phonophobia^3,4^. Previous research has suggested that auditory hypersensitivity is a manifestation of sensitization areas of the brain related to the processing of auditory information^5^. In recent years, there has been increasing focus on auditory and cortical function changes in migraine. However, there are numerous studies with varying methods and inconsistent conclusions. This may be attributed to the mild nature of hearing damage in migraine patients and the lack of obvious cortical function changes.

Event-related potential (ERP) is a bioelectrical response detected after a specific stimulus is given to the nervous system, which has a relatively fixed time interval and a specific phase with the stimulus and can reflect the central brain response of the higher cortex of the brain to a specific stimulus. Commonly used auditory event-related potentials include P1-N1-P2 complex wave, mismatch negativity (MMN), and P300. Among them, the MMN is an endogenous ERP elicited by the brain in response to an occasional deviant stimulus interspersed with a set of repetitive standard auditory stimuli. It primarily occurs in the auditory cortex, with the involvement of the bilateral frontal lobe and hippocampus. The MMN typically occurs 100–300 ms after the stimulus and peaks 150–250 ms after the onset of the change, with a shorter latency and increased amplitude as the magnitude of the stimulus change increases^6^. In most studies, standard and deviant stimuli (e.g., tones of 1000 and 2000 Hz, respectively) are typically presented in random order within the paradigms. However, the MMN can also be elicited by complex stimuli, such as alterations in timbre, pitch, intensity, and rhythm^7^. Experiments typically employ the Oddball model for MMN. The emergence of MMN is not contingent on whether the subject pays attention to the sound, as it can manifest even without the subject’s focus^8^. Therefore, as an objective, non-invasive, and reproducible electrophysiological index, the MMN can objectively reflect the automatic processing of auditory information in the brain. This has the potential for application in clinical and cognitive testing and has already been used in cochlear implants for the objective assessment of speech perception experiments^9^.

In this study, we utilized the Hyperacusis Questionnaire and ERP test to examine the auditory hypersensitivity and cortical function of migraine patients. Alterations in latency and amplitude of the MMN and P300 components were assessed. The aim was to contribute to a better understanding of the physiological relationship between migraine and auditory hypersensitivity.

## Information and methodology

### Study objects

Seventeen migraine patients were selected from the outpatient clinic of the Department of Otorhinolaryngology, Head and Neck Surgery at Peking University People’s Hospital from June 2023 to September 2023.

Inclusion Criteria: The study used the International Classification of Headache Disorders, 3rd edition (ICHD-3) migraine diagnostic criteria, which required meeting the following criteria: (1) experiencing at least 5 episodes of headache attacks lasting 4–72 h, whether treated or untreated; (2) having at least 2 of the following headache characteristics: unilateral pain, throbbing sensation, moderate or severe headache, headache worsened by routine physical activities (e.g., walking or climbing stairs), or avoiding such activities during a headache; (3) experiencing headache accompanied by at least 1 of the following: sensitivity to sound, nausea, vomiting, or sensitivity to light.

Exclusion Criteria: (1) Age > 40 or < 18; (2) other primary or secondary headaches, or medications and foods affecting migraine symptoms during the study period and within two weeks; (3) history of organic neurological diseases (e.g., infections, tumors, etc.) or coexisting with other psychiatric disorders (anxiety, depression, etc.); (4) diseases affecting intellectual ability such as Alzheimer’s disease, dementia, etc.; (5) diseases affecting assessment such as visual and articulation disorders; (6) cultural level is lower than university ; (7) Sound discrimination disorders in daily life.

Nineteen healthy individuals without a history of headaches or chronic diseases from the physical examination center were included in the control group. The two groups were matched for age, gender, and education level. The study was approved by the Ethics Committee of the Peking University People’s Hospital. All methods were performed in accordance with the relevant guidelines and regulations by including a statement in the methods section to this effect, and all participants agreed to take part in the trial and provided written informed consent. (Ethical approval number: 2023PHB229-001).

### Research methodology

#### General subject collection

General information about the subjects in the two groups was collected with a questionnaire, which included age, gender, BMI, education level, and medical history. Depression and anxiety issues were ruled out using the PHQ-9 Depression Screening Scale and GAD-7 Generalized Anxiety Self-Rating Scale (PHQ-9 score ≤ 15 and GAD-7 score ≤ 15).

One patient in the migraine group was excluded because he scored 17 on the PHQ-9 Depression Screening Scale. According to the scoring criteria, PHQ-9 > 15 suggests the possibility of major depression, and GAD-7 > 15 suggests the possibility of major anxiety, which requires professional medical counseling^10,11^. Relevant studies have shown that patients with major depression have prolonged MMN latency and reduced amplitude, so they were excluded^12^.

#### Hyperacusis questionnaire assessment and tinnitus handicap inventory

The Hyperacusis Questionnaire and Tinnitus Handicap Inventory was administered to two groups by the same researcher with specialized training^13^. The full questionnaire is available in the Online Appendix.

#### Event related potential (ERP)

Sampling equipment: The stimuli were presented with a Hewlett-Packard desktop computer, and the electroencephalography was recorded synchronously using an 8-channel Nicolet EDX Nicolet EMG evoked potential apparatus and its accompanying software.

Stimulation sequence: The Oddball classical paradigm was used to provide acoustic stimulation. This involved randomly presenting high-frequency pure tones (2000 Hz, deviant stimulus, 80 dB) and low-frequency pure tones (750 Hz, standard stimulus, 80 dB). Each stimulus lasted for 500 ms with a stimulus interval of 1000 ms. The ratio of high-frequency pure tones to low-frequency pure tones was 2:8. The recording equipment automatically excluded brainwave artifacts, and a total of 200 effective stimuli were presented.

Data collection and analysis: reference to previous relevant literature^8^, three electrode points were selected for recording brain activity: the anterior (Fz), middle (Cz), and posterior (Pz) of the brain. These electrodes were placed at the scalp. The ground electrode was positioned in the middle of the forehead (Fpz point), and the reference electrode was located in the ipsilateral postauricular or mastoid. The amplifier was connected to a bandpass filter with a range of 1.0–30.0 Hz, the sensitivity was adjusted to 20 μV, and the impedance was maintained below 15 kΩ. Mis negative wave detection was conducted in a tranquil, shielded room with a temperature ranging from 20 to 25 °C. Participants were directed to relax all their muscles and watch a silent video. All trials of the two experimental conditions (Oddball block standard stimulus, Oddball block deviant stimulus) were combined and averaged to obtain the standard and deviant ERPs. Subsequently, the deviant stimulus ERPs in the Oddball block were subtracted from the standard stimulus ERPs in the Oddball block to derive the third set of ERPs known as MMN. The mean MMN amplitude (averaged across three electrodes) was measured separately for each subject, N1 time window of 100–200 ms, the P2 time window of 200–300 ms, the MMN time window of 150–250 ms, and the P300 time window of 250–450 ms.

### Statistical analysis

SPSS25. 0 statistical software was used. The overall health status, Hyperacusis Questionnaire, MMN and P300 latency and amplitude of the participants in the study were subjected to statistical analysis. Gender was represented as a binomial distribution. Additionally, all the rows of experimental data were tested for normality. Data that followed a normal distribution were described using the mean and standard deviation $\left( {\overline{x} \pm {\text{s}}} \right)$, while the median (quartiles, Q25, Q75) was used for data that did not conform to a normal distribution. An independent samples t-test was used. For non-normally distributed data, a non-parametric rank sum test was used, while the χ^2^ test was employed to assess differences in gender composition. Additionally, Pearson correlation analysis was conducted to examine the relationship between Hyperacusis Questionnaire and MMN components, with a significance level of *P* < 0.05 considered statistically significant.

### Ethical approval

Ethical approval number: 2023PHB229-001 from the Ethics Committee of Peking University People’s Hospital.

## Results

### General information

A total of 17 migraine patients were included in this study, 7 (41.2%) males and 10 (58.8%) females, with an average age of (26.65 ± 6.75) years, BMI of (20.91 ± 3.25) kg/m^2^, PHQ-9 score of (7.06 ± 4.47), GAD-7 score of (6.35 ± 4.47), and a mean disease duration of (8.93 ± 6.63) years. The duration of headache attacks was approximately 5.2 h, and the frequency of headache attacks was about 12–15 times per month. Nineteen participants were included in the control group, comprising 10 males (52.6%) and 10 females (47.4%) with an average age of (27.68 ± 6.15) years, BMI of (23.30 ± 3.14) kg/m^2^, PHQ-9 score of (6.11 ± 3.99), and GAD-7 score of (4.42 ± 3.93). The education level of the patients in both the migraine group and the control group was at least a bachelor’s degree, and there were no statistically significant differences in age, PHQ-9 score, GAD-7 score and education level (*P* > 0.05). The BMI of the migraine group was significantly lower than that of the control group (*P* = 0.032) (Table 1).

**Table 1:** The general condition of migraine and control group ($\overline{x}$ ± s).

Groups	Number	Age	BMI	PHQ-9 score	GAD-7 score
Migraine	17	26.65 ± 6.75	20.91 ± 3.25	7.06 ± 4.47	6.35 ± 4.47
Control	19	27.68 ± 6.15	23.30 ± 3.14	6.11 ± 3.99	4.42 ± 3.93
*P* value		0.633	0.032	0.174	0.177

### Pure tone audiometry (PTA) and auditory brainstem response (ABR)

ABR thresholds for all subjects were < 40 dBnHL. All subjects had no significant abnormalities in all frequencies of PTA. Compared with the control group, there was no statistically significant difference between the PTA, ABR I, III, and V wave latencies and I–V wave intervals, as well as the thresholds in migraine patients (all *P* > 0.05) (Tables 2, 3).

**Table 2:** Comparison of wave I, III, and V latencies and wave I-V intervals of ABR in migraine and control groups ($\overline{x}$ ± s).

Groups	Number	I (ms)		III (ms)		V (ms)		I–V (ms)	
		Left	Right	Left	Right	Left	Right	Left	Right
Migraine	17	1.70 ± 0.12	1.75 ± 0.16	3.85 ± 0.15	3.85 ± 0.15	3.86 ± 0.16	3.86 ± 0.16	4.04 ± 0.21	4.03 ± 0.26
Control	19	1.73 ± 0.18	1.67 ± 0.16	3.94 ± 0.21	3.94 ± 0.21	3.94 ± 0.22	3.94 ± 0.22	4.03 ± 0.29	4.15 ± 0.25

**Table 3:** Comparison of all frequencies of PTA in migraine and control groups ($\overline{x }$±s).

Groups	Number	250 Hz (dB)		500 Hz (dB)		1000 Hz (dB)		2000 Hz (dB)		4000 Hz (dB)		8000 Hz (dB)	
		Left	Right	Left	Right	Left	Right	Left	Right	Left	Right	Left	Right
Migraine	17	3.85 ± 3.63	4.23 ± 4.00	4.23 ± 4.00	5.00 ± 5.00	5.00 ± 6.12	5.38 ± 5.94	3.08 ± 4.35	4.23 ± 4.49	3.85 ± 3.63	5.00 ± 4.08	13.85 ± 21.91	11.25 ± 14.00
Control	19	6.33 ± 4.42	4.33 ± 3.20	8.00 ± 5.92	6.33 ± 4.42	8.33 ± 6.17	6.33 ± 3.99	7.33 ± 5.94	6.33 ± 3.99	5.67 ± 4.95	5.33 ± 3.99	10.33 ± 8.76	10.33 ± 9.90

### Hyperacusis questionnaire and tinnitus handicap inventory

The score on the Hyperacusis Questionnaire was 12.47 ± 6.49 in the migraine group and 5.89 ± 6.51 in the control group. The score in the migraine group was significantly higher than that in the control group (*P* = 0.005). In the migraine group, one had mild tinnitus disorder and two had moderate tinnitus disorder; in the control group, two had mild tinnitus disorder, one had moderate tinnitus disorder, and another one had severe tinnitus disorder. The score on the Tinnitus Handicap Inventory was 11.71 ± 18.36 in the migraine group and 11.22 ± 21.23 in the control group (*P* > 0.05). In the migraine group, the correlation coefficient between the Hyperacusis Questionnaire and the THI was 0.158 (*P* > 0.05), In the control group, the correlation coefficient was − 0.217 (*P* > 0.05). The number of patients with migraine who experienced tinnitus was low in their cohort, and hyperacusis was not related to tinnitus in individuals with migraine.

### Comparison of MMN and P300 latency and amplitude between the two groups

In the migraine group, the MMN latency was (148.82 ± 22.3) ms and (150.18 ± 22.72) ms at the Fz and Cz points. The amplitudes were (7.97 ± 3.98) μV and (7.66 ± 4.56) μV. In the control group, the MMN latency was (177.37 ± 17.41) ms and (179.53 ± 18.33) ms at the Fz and Cz points. The amplitudes were (4.70 ± 1.36) μV and (4.47 ± 1.65) μV. P300 latency in the migraine group was (329.25 ± 43.24) ms and (326.94 ± 47.10) ms at the Cz and Pz points, with amplitudes of (3.55 ± 2.54) μV and (3.82 ± 2.95) μV. The P300 latency in the control group was (339.95 ± 37.78) ms and (341.74 ± 40.93) ms at the Cz and Pz sites. The amplitudes were (3.94 ± 2.42) μV and (3.14 ± 2.09) μV. At the Fz and Cz sites, the MMN latency was significantly shorter in the migraine patients compared with the control group (P values were < 0.0001), and the amplitude was significantly higher (*P* values were < 0.05, 0.004, and 0.013, respectively). However, there was no statistically significant difference in the P300 latency and amplitude between migraine patients and the control group (both *P* > 0.05). Refer to Table 4.

**Table 4:** Comparison of latency and amplitude of MMN and P300 in migraine and control ($\overline{x }$±s).

Groups	Number	MMN-FZ		MMN-CZ		P300-FZ		P300-CZ	
		Latency (ms)	Amplitude (μV)	Latency (ms)	Amplitude (μV)	Latency (ms)	Amplitude (μV)	Latency (ms)	Amplitude (μV)
Migraine	17	148.82 ± 22.3	7.97 ± 3.98	150.18 ± 22.72	7.66 ± 4.56	329.25 ± 43.24	3.55 ± 2.54	326.94 ± 47.10	3.82 ± 2.95
Control	19	177.37 ± 17.41	4.70 ± 1.36	179.53 ± 18.33	4.47 ± 1.65	339.95 ± 37.78	3.94 ± 2.42	341.74 ± 40.93	3.14 ± 2.09
*P* value		< 0.0001	0.004	< 0.0001	0.013	0.440	0.644	0.327	0.435

### Correlation Analysis of Hyperacusis Questionnaire with Latency and Amplitude of MMN and P300

**Figure 1:**
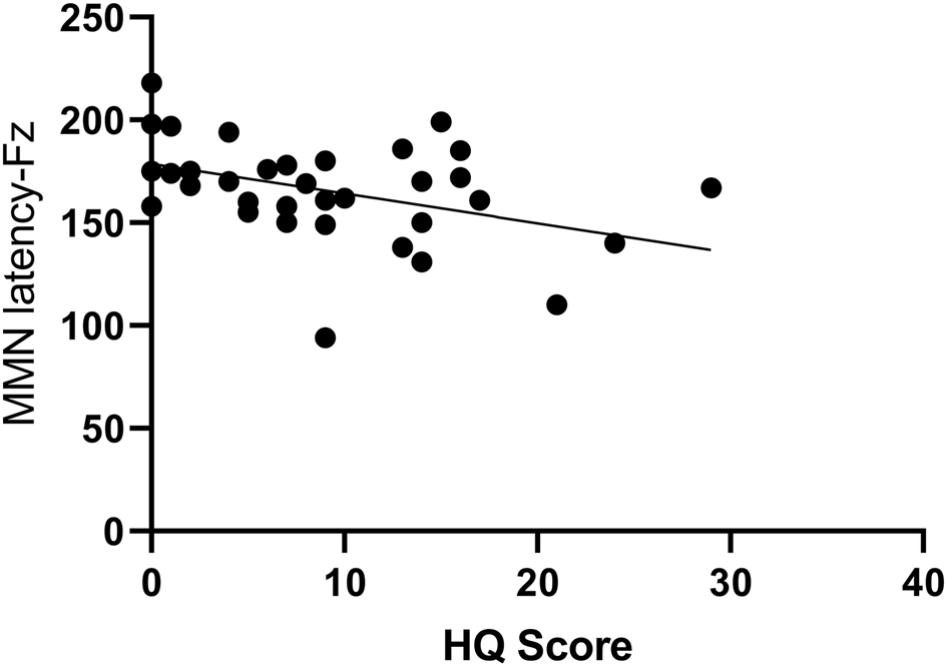
The Hyperacusis Questionnaire negatively correlated with MMN (Fz) latency (*P* < 0.05).

**Figure 2:**
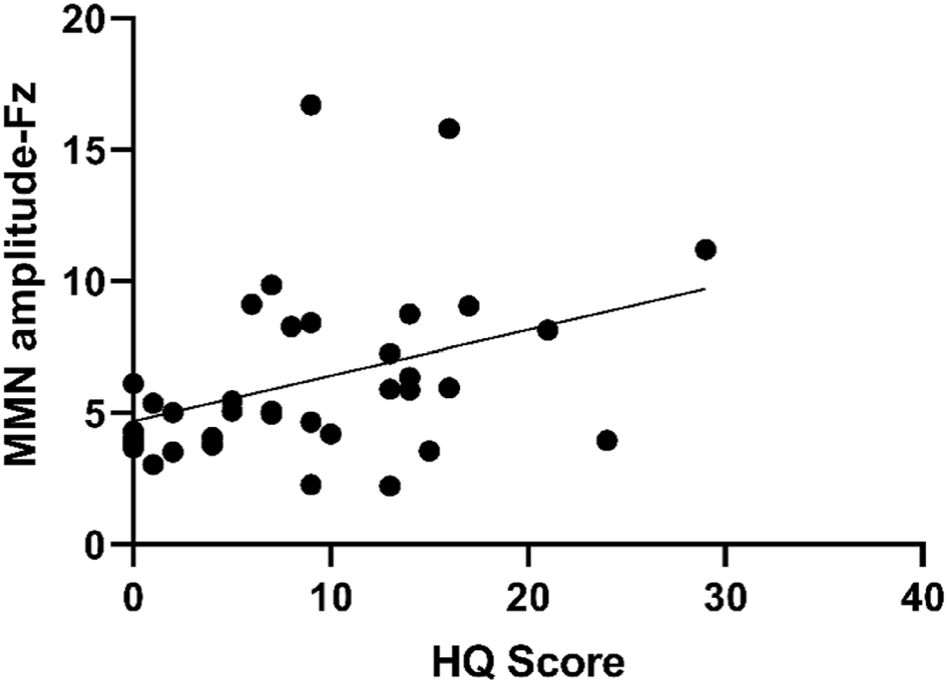
Positive correlation between Hyperacusis Questionnaire and MMN (Fz) amplitude (*P* < 0.05).

The correlation coefficient between the Hyperacusis Questionnaire and the MMN latency at point Fz was − 0.374 (*P* = 0.025), indicating that the auditory hypersensitivity score was negatively correlated with the MMN latency. Refer to Fig. 1. The correlation coefficient between the Hyperacusis Questionnaire and the MMN amplitude at point Fz was 0.378 (*P* = 0.023), indicating a positive correlation between the auditory hypersensitivity score and the MMN amplitude. Refer to Fig. 2. There was no significant correlation between the Hyperacusis Questionnaire and P300 latency and amplitude (*P* > 0.05).

## Discussion

The pathogenesis of migraine is inconclusive, and the prevailing view is the neurovascular theory, particularly the brainstem-trigeminal-vascular reflex theory. Migraines who had exposed to internal and external environmental stimuli, the trigeminal nucleus is connected to the contralateral thalamus, which then sends projections to the temporal and parietal lobes, as well as to cortical regions of the insula and cingulate gyrus. When trigeminal nerve endings are stimulated, vasoactive peptides are released, leading to neurogenic inflammation and sensitization of the trigeminal nerve. This can result in hyperexcitability, headaches, and abnormal psychosomatic and emotional responses, even with mild chemical or mechanical stimuli^14^. Patients with migraine are often accompanied by anxiety, irritability, depression, and other psychosomatic symptoms^15^. The present study compares migraine patients with non-migraine counterparts. In this study, we compared anxiety and depression ratings between migraine patients and healthy individuals. We found that migraine patients had higher anxiety scores, but the differences were not statistically significant.

Auditory hypersensitivity is commonly described as phonophobia. It can occur during both the prodromal and episodic phases of a migraine attack. As early as 1984, Kayan et al. found that 81% of migraineurs experienced phonophobia during headache attacks^4^. Later, Bhola et al. used single-pulse transcranial magnetic stimulation to treat 190 migraine patients and found that after depolarizing specific areas of the brain, 62% of the patients reported a reduction in headache symptoms, while 53% of the subjects showed a reduction in phonophobia. This suggests that pain and auditory sensitivities in migraine patients may be related to cortical hyperexcitability^3^. The results of our study show that migraine patients scored higher on the Hyperacusis Questionnaire compared to the control group. This is consistent with the clinically observed phenomenon. Auditory hypersensitivity symptoms are strongly associated with the severity of headaches and have been shown to improve with migraine prophylaxis^16,17^. Previous studies have concluded that hyperacusis does not contribute to greater tinnitus burden when tinnitus is severe^18^. In our study, the number of patients with migraine who experienced tinnitus was low in their cohort, and THI scores are not related to HQ scores in the migraine group and the control group, which confirms previous studies.

Event-related potentials (ERPs) are a non-invasive and easy-to-implement neurophysiological technique that can demonstrate the cognitive processes of brain nerve cell activity in millisecond units by recording neurophysiological changes on the surface of the scalp while the patient is awake. It offers the benefits of high temporal resolution, minimal requirements for patient cooperation, ease of operation, and is not affected by subjective factors such as education level and language. Studies have reported that event-related potentials can provide a more objective reflection of cognitive processing, which is valuable in the context of Alzheimer’s disease, vascular cognitive disorders, and other related conditions^19,20^. The current study utilized auditory event-related potentials to assess cognitive processing. The auditory event-related potential MMN used in this study mainly occurs in the auditory center and frontal cortex, receiving direct input from sensory stimuli. It is very sensitive to behavioral stimuli and attentional shifts, and changes when the brain is impaired in categorizing the stimuli. This is manifested as prolonged latency and reduced amplitude of the MMN. On the other hand, P300 reflects cognitive processes such as memory and the auditory discrimination. The prolongation of its latency suggests a delay in auditory discrimination dysfunction, and may related to memory dysfunction, while the reduction of amplitude reflects impaired attention, which is a component of executive function^20,21^. In summary, the MMN reflects the speed of sound stimulus classification in the subjects’ brains, while the P300 reflects cognitive functions such as memory and attention. Therefore, in this study, the MMN and P300 examinations were combined with audiological tests to analyze auditory hypersensitivity symptoms in migraine patients and investigate changes in auditory center function.

Previous studies have shown that individuals with migraines exhibit a heightened attentional response to visual stimuli^22,23^. Neuroimaging studies suggest that this may be due to a weaker brain response to stimulus changes in migraineurs, leading to ineffective processing of different stimuli^24^. For example, individuals with migraines exhibited an increase in the amplitude of visual stimuli in response to a recurring pattern of oddballs, while the control group showed a gradual decrease^25^. Therefore, we hypothesized that individuals with migraines would also exhibit heightened cortical activity, such as increased amplitude in auditory Oddball pattern tests. According to previous studies^8^, the MMN waveform is most pronounced at the Fz point, and the P300 waveform is most pronounced at the Pz point. Therefore, the Fz and Cz points were selected for analyzing the MMN data, and the Cz and Pz points for analyzing the P300 data. In this study, the MMN latency at the Fz and Cz sites of migraine patients was significantly shorter (*P* < 0.001), and the amplitude was significantly higher (*P* < 0.05) than those of the control group. This suggests that migraine patients process the acoustic stimuli faster and exhibit increased cortical excitability, supporting the symptom of abnormal sensitivity to noise in migraine patients. These findings are consistent with previous studies^23,26–28^. The differences in P300 latency and amplitude at Cz and Pz sites in migraine patients were not statistically significant (*P* > 0.05) compared to those of the control group, indicating that there may be less impairment in cognitive function in migraine patients. However, due to testing conditions, we did not perform cognitive function tests such as attention and memory neither auditory discrimiation tests on the subjects to further validate cognitive function changes in migraine patients. Furthermore, the study’s findings revealed a notable correlation between MMN and Hyperacusis Questionnaire scores. Auditory hypersensitivity scores were found to have a negative correlation with MMN latency and a positive correlation with MMN amplitude. This confirms that MMN serves as an objective neurophysiological index for evaluating auditory hypersensitivity symptoms in migraine patients.

In future studies, the sample could be increased, and migraine patients could be further categorized based on the presence or absence of aura and whether they are in the attack phase. An objective auditory hypersensitivity assessment could be employed, and subjects could be followed up to observe the dynamic changes in auditory hypersensitivity symptoms and cognitive function.

### Supplementary Information

Supplementary Information 1.

## Data Availability

The datasets generated during and/or analysed during the current study are available from the corresponding author on reasonable request.
