# Supplementary figures and images for: Hyperacusis questionnaire and event-related potential correlation in migraine patients
Source: Sci Rep. 2024 Jun 19;14:14117. doi: 10.1038/s41598-024-65014-3 (PMC11187201; doi:10.1038/s41598-024-65014-3)

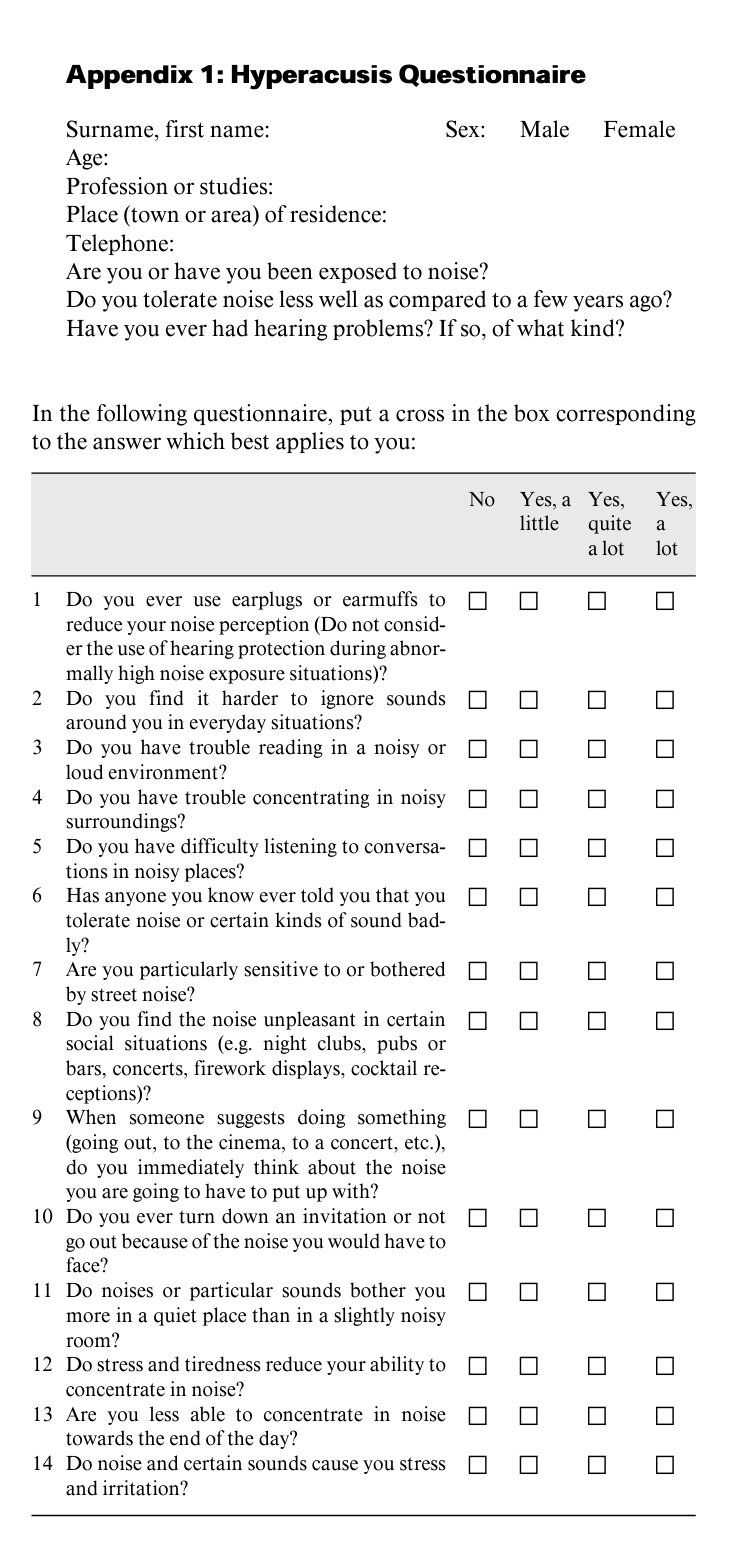

Supplement: Supplementary file 1 — Supplementary Information 1. [file 41598_2024_65014_MOESM1_ESM.png]
